# Stage-Dependent Persistence of Nucleated Endosperm Cells in Seeds of *Limonium* Sexual and Apomictic Species with Autonomous Endosperm Formation

**DOI:** 10.3390/genes17030337

**Published:** 2026-03-18

**Authors:** Catarina Gomes-Domingues, Elvira Hörandl, Ana D. Caperta

**Affiliations:** 1 Linking Landscape, Environment, Agriculture and Food (LEAF) Research Center, Associate Laboratory TERRA, Instituto Superior de Agronomia (ISA), Universidade de Lisboa, Tapada da Ajuda, 1349-017 Lisboa, Portugal; catarinaagomes01@gmail.com; 2 Department of Systematics, Biodiversity and Evolution of Plants (with Herbarium), University of Göttingen, 37073 Göttingen, Germany; ehoeran@uni-goettingen.de

**Keywords:** apomixis, seed phenology, single-seed flow cytometric screening, reproduction mode, morphological markers

## Abstract

*Limonium* Mill. species present a polymorphic sexual system associated with flower polymorphisms like ancillary pollen and stigma, with sexual and/or apomictic reproduction. The aim of this study was to investigate the reproductive traits, test for autonomous apomixis, and assess seed formation in triploid *Limonium algarvense* and *Limonium daveaui*. Pollen-stigma combinations were determined and the number of flowers and seeds counted. Single-seed flow cytometry was performed using seeds in three phenological stages: immature (stage I), early maturing (stage II) and mature seeds (stage III). The findings revealed that all triploid plants were self-sterile and produced seeds in the absence of pollination. Despite *L. daveaui* having a higher number of flowers than *L. algarvense*, a significantly higher ratio of seeds/flowers was observed in the latter species. Stage-dependent endosperm developmental patterns were observed, with nucleated cells present in stage II seeds with a light brown or pinkish coat, and an embryo peak and an endosperm peak with the double ploidy level. Stage III seeds, with a dark brown coat, presented only an embryo peak. Additionally, a single hexaploid endosperm peak was detected in stage I seeds, revealing early initiation of the endosperm with nucleated cells prior to embryo development. The single 6C endosperm peak was always associated with shrunken and wrinkled or underdeveloped stage I seeds but was never detected in stage II seeds. Overall, our results support reproduction via asexually formed seeds with pollen-independent endosperm formation and allow the identification of phenological development stages and seed coat morphological markers associated with single-seed flow cytometric screening patterns in apomictic species.

## 1. Introduction

Apomixis is an asexual reproductive strategy in plants in which seeds form without fertilization [1]. The principal developmental pathways of apomixis are gametophytic and sporophytic [2,3,4]. In gametophytic apomixis, an unreduced embryo sac forms either through apospory, originating from a somatic nucellus cell, or through mitotic diplospory, arising from a restitutional meiotic process. This unreduced egg cell forms an embryo without fertilization via parthenogenesis. In gametophytic apomicts, male gametes are generally fundamental for fertilization of the polar nuclei in the embryo sac, ensuring proper endosperm development (pseudogamy, [1]). In general, the apomictic pathways affect only female development, while male meiosis and microgametogenesis are not altered, resulting in gametes with reduced chromosome number [5,6]. Proper endosperm development depends on maintaining the correct balance between maternal and paternal chromosomal contributions, which typically requires a 2:1 maternal-to-paternal genome ratio (2m:1p) [7]. However, some populations or apomictic species do not follow this rule, producing viable seeds with other than 2m:1p and forming autonomous endosperm [8,9].

In some flowering plant families, apomixis with pollen-independent autonomous endosperm formation is found as in Asteraceae polyploid apomicts (*Taraxacum officinale*, [10,11], *Hieracium* [12], *Erigeron* [10,13]), but is rarely observed in diploids [10,13]. The same applies to the genus *Limonium* Mill. (Plumbaginaceae), at least for triploid and tetraploid apomicts [14,15], in which the endosperm is rapidly consumed [15,16] as seen in Asteraceae [10]. In apomictic *Hieracium*, pollen production is heterogeneous, and viability is variable, with many apomicts being completely pollen-sterile while others produce less pollen compared to sexual diploids [17,18]. In *Limonium*, male abortion was observed in some tetraploid apomicts (2*n =* 4*x* = 35, 36 chromosomes) that form a high seed set in the absence of pollination [15,16]. Triploid *Limonium algarvense* plants (2*n =* 3*x* = 25, 26) show disturbed male meiosis due to pairing and segregation anomalies, and thus produce a high frequency of inviable aneuploid pollen grains but retain residual male fertility [19].

The genus *Limonium* exhibits considerable diversity of floral traits and reproductive systems, encompasses ca. 600 species [20,21,22,23,24,25,26,27,28,29,30,31,32], and is characterized by a high incidence of polyploidy [21,27,33,34,35]. Plants show a polymorphic sexual system associated with striking flower polymorphisms linked to a sporophytic self-incompatibility system [15,20,36,37]. Flowers can show four pollen-stigma combinations, respectively: A—coarse reticulate sexine (pollen type A) and a cob-like stigmatic papillae (A/cob); B—finely reticulate sexine and a papillate type stigma (B/Pap); C—finely reticulate sexine—cob type stigma (B/Cob); and D—coarsely reticulate sexine—papillate type stigma (A/Pap) [20]. Dimorphic diploid and polyploid species with even chromosome numbers and A/cob and B/papillate flowers have been considered to reproduce through outcrossing, whereas monomorphic species that exhibit self-compatibility (typically A/Pap, though B/cob occurs less frequently) seem to reproduce through selfing [20,21,38]. Monomorphic aneuploid and polyploid species with A/cob or B/papillate and odd chromosome counts were considered apomicts [6,33,37].

Embryological investigations in *Limonium* show that sexual taxa produce reduced embryo sacs of tetrasporic (meiotic) origin, corresponding to the *Gagea ova* type [39] or to the *Adoxa* and *Drusa* types [15,40]. Tetraploid apomictic, male sterile plants (*Limonium multiflorum*) reproduce through mitotic diplospory with *Rudbeckia*-type embryo sacs, although diploids *(Limonium ovalifolium)* may present a low expressivity of apomixis [15]. Facultative gametophytic apomicts like triploid *Statice oleifolia* var *confusa* (=syn. *Limonium virgatum*, 2*n =* 3*x* = 27) form meiotic tetrasporous reduced *Adoxa*-type embryo sacs in parallel with diplosporous development of the *Ixeris* and *Eryngium* types [14,41].

Aneuploidy increases or decreases in specific chromosomes can disrupt gene dosage balance and negatively affect gametophyte development [42,43], causing severe fertility reduction in triploid plants [44]. *Taraxacum* plants are intolerant to aneuploidy [45,46], as shown by apomictic triploid hybrids produced in crosses between sexual diploid and triploid apomicts [47], where nuclear restitution (diplospory) seems to be incomplete, and embryo sacs degenerate before anthesis and appear to lack autonomous endosperm development [48]. Since triploid *Limonium* species represent the dominant cytotype across the Iberian Peninsula and the Balearic Islands [21,33,34,49], these species seem to tolerate aneuploidy, at least eutriploids (triploids possessing three complete chromosome sets [50]).

Large-scale screening techniques like flow cytometric seed screening (FCSS) can be used to detect reproductive modes in flowering plants, including apomixis [51]. This method enables simultaneous assessment of apomeiosis and parthenogenesis, thereby providing insight into functional seed formation. In cases of autonomous apomixis, the FCSS profiles can be challenging to interpret because the resulting embryo:endosperm ploidy ratio of 1:2 produces histogram patterns that overlap with G2 peaks of mitotically active embryonic tissues [6].

This study on *Limonium* plants, representative of diploid and triploid species, addressed the following specific questions: (1) Are there differences in flower opening in relation to the start of seed formation? (2) Do floral combinations relate to sexual and/or apomictic reproduction? (3) What are the endosperm development patterns in immature and mature seed stages? (4) Can FCSS reliably detect apomictic versus sexual seed formation in *Limonium*? To address these questions, we analyzed the reproductive outputs and compared staged seeds from *L. algarvense* and *L. daveaui* with mature seeds from *L. ovalifolium* through single-seed FCSS (ssFCSS).

## 2. Materials and Methods

### 2.1. Plant Material and Growth Conditions

The plants investigated in this study originated from seeds collected in natural populations. *Limonium daveaui* seeds were obtained from saltmarsh populations in Fundação do Samouco salterns complex (Alcochete, Portugal), *L. algarvense* seeds were collected in Guadiana estuary (Algarve, Portugal). Briefly, after germination on water-soaked filter paper as described in [16], seedlings were grown in *jiffy* pots (peat) under controlled conditions (temperature 26 °C/22 °C with 16 h light/8 h dark photoperiod, respectively) in a growth chamber (Rumed, Laatzen, Germany). After three months, seedlings were transplanted into plastic pots with a mixture of autoclave-sterilized (1 h at 120 °C) peat and perlite (1:2 *v*/*v*). Six plants of each triploid species, in a total of twelve plants, were obtained and maintained under greenhouse-controlled conditions at the Instituto Superior de Agronomia (ISA), University of Lisbon, Portugal. From these individuals, leaves and inflorescences of different ages were harvested for flow cytometric analysis. These plants are characterized by indeterminate inflorescences, in which the main axis continues to grow and produce flowers, with the youngest flowers found at the apex [21].

The *L. ovalifolium* plants utilized in this study show inflorescences branched into corymbs [21] and presented self-incompatible flower morph B (papillate stigma and finely reticulate exine) [15]. These plants produced more than a hundred seeds per inflorescence [52].

### 2.2. Floral Heteromorphisms, Flower and Seed Production

A total of three fresh flowers per plant were used for floral heteromorphism determinations in *L. daveaui* and *L. algarvense*, following [15,52]. In brief, flowers were dissected to isolate pollen and stigma, and preparations were observed under a Leica DM500 light microscope (Leica Microsystems, Heerbrugg, Switzerland) with 40× magnification. Stigma and pollen types (A/B pollen and cob-like/papillate stigmas [20]) were determined, and plants were classified as self-compatible or self-incompatible. To test for autonomous apomixis, immature inflorescences were covered with fine mesh bags prior to anthesis.

**Figure 1:**
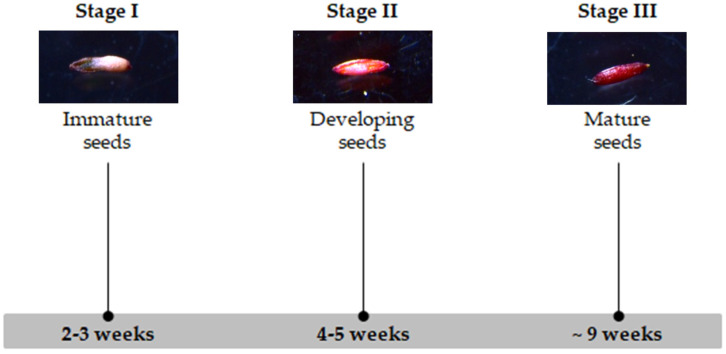
Phenological stages (Stage I–III; 2–9 weeks) of *Limonium* seeds used in this study.

Fructifying scapes with approximately two to four weeks after anthesis were harvested, and flowers and seeds were counted. The proportion between the number of seeds and the total number of dried flowers (including empty flowers) was calculated considering isolated seeds/flowers × 100 (%). Seed set (proportion of well-developed seeds from all seeds counted per plant) was also considered. Inflorescences were separated according to distinct developmental stages, based on the number of days after anthesis, and stored at 4 °C until flow cytometry analysis. Isolated seeds were staged following three development stages (Figure 1): stage I—immature seeds with approximately two to three weeks; stage II—developing seeds within four to five weeks; stage III—mature seeds with around nine weeks. Mature seeds from *L. ovalifolium* inflorescences with five (stage II) and nine weeks (stage III) conserved at −20 °C were also utilized [52]. Seed characterization, namely seed coat appearance and color, and seed sectioning to assess the presence of endosperm in immature seeds, was performed under a Leica M125 stereomicroscope (Leica Microsystems, Wetzlar, Germany).

### 2.3. Somatic Ploidy Estimation and Reproductive Mode Determination

Leaf material from each triploid species was collected, and somatic ploidy measurements were carried out using flow cytometry for each individual separately. Approximately 5 to 10 mm^2^ of *Limonium* leaf tissue was chopped with a razor blade [53] together with silica-dried leaf material of the internal reference standard *Pisum sativum* ‘Ctirad’ (2*C* = 9.0 pg) [54]. Nuclei were isolated following the protocol described in [55]. The results were obtained using a CyFlow Ploidy Analyzer (Sysmex, Norderstedt, Germany) in conjunction with CUBE16 v.1.6 software (Sysmex, Norderstedt, Germany). Deionized water (dH_2_O) was used in place of sheath fluid. The measured median size of intact nuclei (within a target region, or ‘peak’) was compared to the nuclei size of the internal reference. Prior to analysis, ploidy inference of the diploid *L. ovalifolium* “Lo2009I2CR” [56] was confirmed. Samples with CV values of G1 peaks that overpass the established threshold (<5%) were discarded. The value of genome size in mass units (2C in pg; sensu [56,57]) was obtained using the following equation: *Limonium* 2C nuclear DNA content (pg) = (*Limonium* G1 peak mean/reference standard G1 peak mean) × genome size of the reference standard [56].

To determine whether individual seeds were produced via sexual reproduction or apomixis, we conducted single-seed flow cytometric seed screening (ssFCSS) [51]. For extracting the nuclei from the seed tissue, a previously described protocol was followed [55] with slight modifications. Each seed was chopped individually with a razor blade in 1 mL of lysis solution and then filtered through 30 µm mesh to obtain a suspension of nuclei. The flow cytometer was flushed with dH_2_O between samples to prevent carryover. The DNA content (relative fluorescence intensity) was calculated with CUBE16 v.1.6 software (Sysmex, Norderstedt, Germany) based on the median peak values corresponding to embryo and endosperm nuclei. Seeds from diploid *L. ovalifolium* (2*C* = 3.53 pg) [26] were used as both internal and external standard (performed prior to each measurement series) to compare median embryo and endosperm nuclei genome sizes and infer ploidy levels. To determine the reproduction mode, we calculated a peak index (PI) metric, which corresponds to the ploidy of the endosperm tissue divided by that of the embryonic tissue. We conducted ssFCSS analysis for 15 seeds per plant and six plants per triploid species, in a total of 180 ssFCSS measurements.

### 2.4. Chromosome Preparations and Counts

Chromosomes were counted for five *L. daveaui* plants obtained from plants collected in the Alcochete population previously mentioned, following [26]. Briefly, root tips were excised and cold-treated for 36 h at 0 °C. Afterwards, root tips were fixed in a fresh absolute ethanol:glacial acetic acid (3:1, *v*/*v*) solution overnight and stored in 70% ethanol at −20 °C. Root tips were then digested in a pectolytic enzyme mixture (2% cellulase [Sigma, St. Louis, MO, USA], 2% cellulase “Onozuka R10” [Serva, Heidelberg, Germany], and 2% pectinase enzyme [Sigma, Lisboa, Portugal] solution in 1× Enzyme Buffer [EB, 40 mL 0.1 M citric acid1hydrate and 60 mL of 0.1 M sodium citrate dihydrate; pH 4.8]) for 150 min at 37 °C. Chromosome preparations were made in 60% acetic acid and stained with 4′,6-diamidino-2 phenylindole hydrochloride (DAPI; 1 mg/mL). Chromosomes were observed and counted under a Zeiss Axioskop 2 fluorescence microscope and photographed with an AxioCam MRc5 digital camera (Zeiss, Göttingen, Germany).

### 2.5. Statistical Analysis

For all data, normality and homogeneity of variances were tested prior to analyses using the Shapiro–Wilk test and Levene’s test, respectively. Differences between triploid species in the number of seeds and flowers produced, as well as the ratio between them, were assessed using a parametric *t*-test or a non-parametric Mann–Whitney U test. Data visualization was carried out through boxplots. Statistical analyses were performed using statistical software R Studio version 4.4.0 for Windows.

## 3. Results

### 3.1. Genome Size, DNA Ploidy Estimations and Chromosome Numbers

Flow cytometric analysis using leaf material revealed a mean fluorescence ratio of 0.39 relative to the internal standard *P. sativum* (2C = 9.0 pg), confirming a diploid genome for *L. ovalifolium* with an inferred 2C = 3.53 pg. Mean fluorescence ratios of 0.57 and 0.62 relative to the internal standard were obtained for *L. daveaui* and *L. algarvense*, corresponding to inferred 3C genome sizes of approximately 5.14 pg and 5.67 pg, respectively.

**Figure 2:**
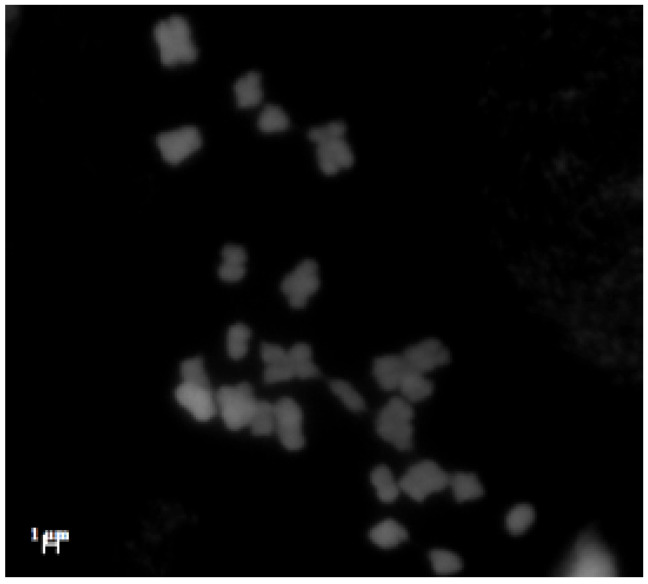
*Limonium daveaui* mitotic metaphase plate of DAPI-stained metaphase spreads (2*n* = 3*x* = 25 chromosomes). Scale bar = 1 μm.

Chromosome counts were made on micrographs of mitotic metaphase spreads of three *L. daveaui* specimens, and, for the first time, 2*n* = 3*x* = 25 chromosomes were revealed for this species (Figure 2). Chromosome numbers for *L. ovalifolium* (2*n* = 2*x* = 16 chromosomes; [16]) and *L. algarvense* (2*n* = 3*x* = 25; [21,22,26]) have been previously described.

### 3.2. Reproductive Analysis

In both triploid species five stigmas with papillate cells and pollen with microreticulate exine were observed. Plants presented the pollen-stigma combination B/pap, indicating self-incompatibility, but seeds were formed in the absence of pollination.

In terms of reproductive development, no significant differences were found in the number of flowers and seeds among plants (Table 1). Although *L. daveaui* produced more flowers, *L. algarvense* developed more inflorescences and had higher mean seed values than the first species.

**Figure 3:**
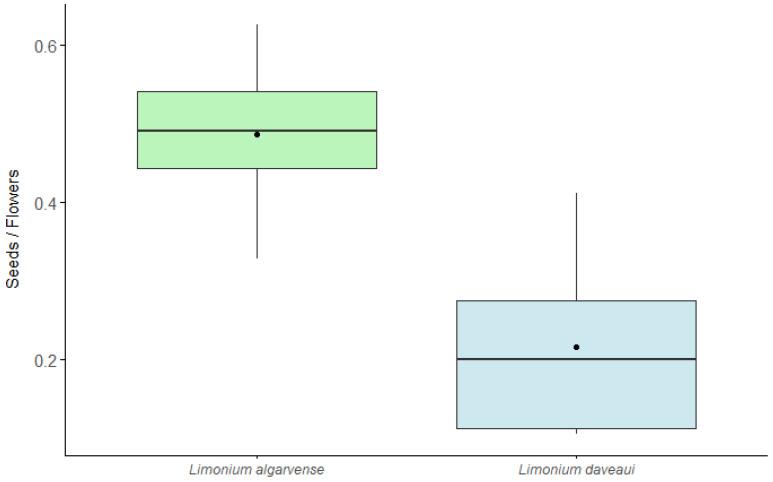
Proportion of seeds produced considering the total number of flowers (seeds/flowers) in the studied triploid *Limonium* plants. In the boxplots, boxes represent the interquartile range (25th–75th percentiles), the central line is the median, the whiskers extend to 1.5× interquartile range, and black points indicate means.

On the other hand, the proportion between the number of seeds and the total number of dried flowers (seeds/flowers) differed significantly between triploid species (independent-samples *t*-test: *t* = 4.096, *p* = 0.002). *Limonium algarvense* showed a higher seeds/flowers ratio (mean = 0.49) than *L. daveaui* (mean = 0.22), with the latter species exhibiting a greater number of empty flowers and aborted seeds. The boxplot (Figure 3) displays the distribution of the data.

### 3.3. Reproductive Modes

**Figure 4:**
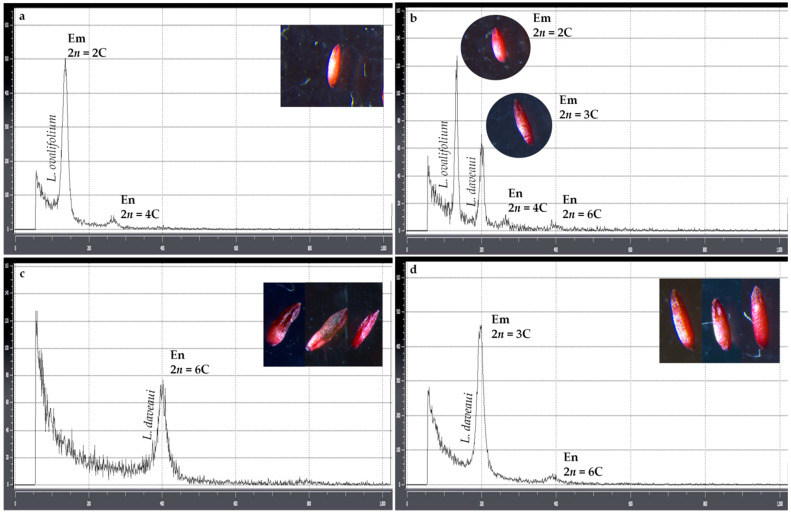

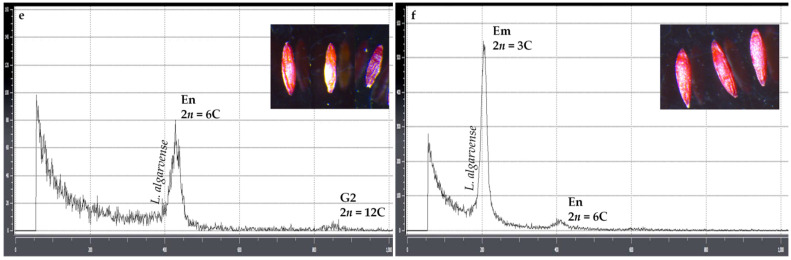
Flow cytometric histograms of DAPI-stained nuclei of *Limonium* seeds. Diploid *Limonium ovalifolium* with sexual reproduction (**a**,**b**), and triploids *Limonium daveaui* (**b**–**d**) and *Limonium algarvense* (**e**,**f**) with autonomous apomixis, at different developmental stages. Diploid plants with diploid embryo and tetraploid endosperm peak originated from a tetrasporic embryo sac. Triploid plants with triploid embryo and hexaploid endosperm peak originated from a diplosporic embryo sac. (**a**) Stage II seed of *L. ovalifolium* used as external standard, with big 2C embryo peak and small 4C endosperm peak; (**b**) stage II seed of *L. daveaui* (with 3C embryo and 6C endosperm) together with a diploid seed used as internal standard; (**c**) stage I seed of *L. daveaui* with 6C endosperm peak; (**d**) stage II seed of *L. daveaui* with 3C embryo peak and 6C endosperm peak; (**e**) stage I seed of *L. algarvense* (with 6C endosperm peak, the second peak being a G2 peak of the growing tissue); (**f**) stage II seed of *L. algarvense*, with 3C embryo peak and 6C endosperm peak. At the top of each peak the ploidy level of the embryo and endosperm is indicated. The images included in each histogram represent examples of seeds that gave the corresponding ploidy pattern of embryo:endosperm. The Y-axis was limited to 600 events for Stage II seeds (**a**,**d**,**e**), and to 160 events for Stage I seeds (**d**,**f**) and samples with internal standards (**b**) to minimize background noise and improve visual clarity. Abbreviations: Em—embryo; En—endosperm.

In diploid *L. ovalifolium* seeds, the estimation of embryo and endosperm nuclear DNA content by ssFCSS showed histograms with a diploid embryo peak (mean fluorescence ratio of 0.39 relative to *P. sativum* [2C = 9.0 pg], and inferred 2C = 3.53 pg) and a tetraploid endosperm peak with the double DNA content (mean fluorescence ratio of 0.76 corresponding to inferred 4C = 6.94 pg; Figure 4a,b) in Stage II seeds, and histograms with a single DNA peak representing the diploid embryo in Stage III seeds. The ratio of embryo to endosperm DNA content of 2C:4C produces a peak index (PI) of ~2 (Table 2).

**Figure 5:**
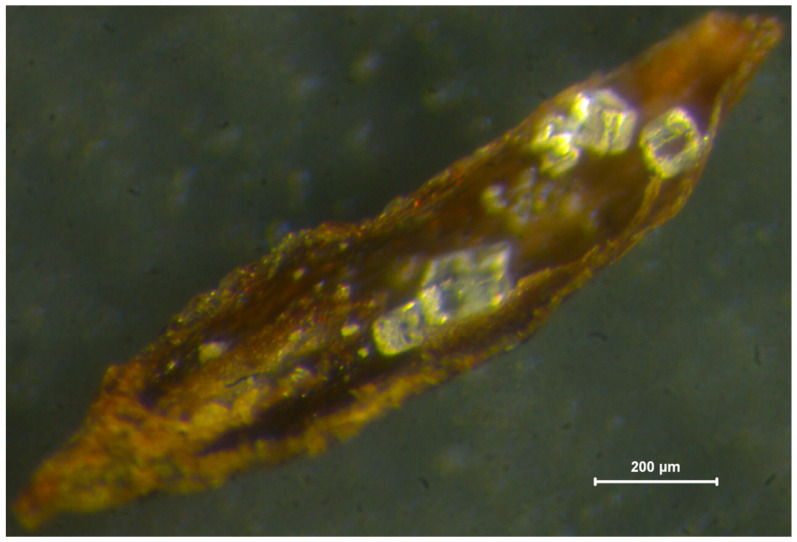
Section of an aborted triploid seed of *Limonium daveaui* (Ld2023I5cs) without a clearly visible embryo but showing endosperm starch grains. Scale bar = 200 μm.

The results of ssFCSS confirmed an apomictic pathway in the triploid plants of both *L. daveaui* and *L. algarvense*. In these species, three different embryo and endosperm ploidy patterns were detected: (i) a single hexaploid endosperm peak obtained from Stage I seeds (mean fluorescence ratios of 2.90 and 3.17 relative to *L. ovalifolium* [2C = 3.53 pg], and inferred nuclear DNA content of approximately 10.23 pg/6C and 11.20 pg/6C for *L. daveaui* and *L. algarvense*, respectively; Figure 4c,e); (ii) two peaks corresponding to a triploid embryo (mean fluorescence ratios of 1.48 and 1.63, and inferred 3C = 5.23 pg and 3C = 5.75 pg for *L. daveaui* and *L. algarvense*, respectively) and an endosperm with a double ploidy level (6C) in Stage II seeds (Figure 4d,f); and (iii) a single triploid embryo peak (3C) observed in Stage III seeds. By visually inspecting seed appearance, differences in seed morphology were seen between seed stages and the associated ssFCSS patterns. In both species, seeds at stage I with a single 6C endosperm peak were shrunken and wrinkled or underdeveloped compared to older seeds with a 3C embryo peak, which were larger and fully developed (Figure 4). Seeds at stage III with a unique 3C peak showed a dark brown coat, while those in stage I and II were pinkish or reddish light brown. Occasionally, aborted seeds were found in both triploids (Figure 5).

Overall, among the 180 seeds analyzed, the proportions of each embryo and endosperm peaks were different. In *L. daveaui*, 20% of the seeds showed a histogram pattern with only an endosperm 6C peak, and 23% a unique embryo 3C peak. While in 57% of the seeds we observed both peaks and the ratio of embryo to endosperm DNA content of 3C:6C, with a peak index (PI) of 2. Similarly, in *L. algarvense* 17% and 22% of the seeds had a histogram with a single 6C or 3C peak, respectively, and in 61%, both 3C and lower 6C peaks were detected. In addition, in some measurements, a smaller endosperm G2 peak was also seen in stage I seeds (Figure 4e).

## 4. Discussion

The ability to produce genetically identical progeny via seed (apomixis) and therefore rapidly fix desirable genotypes is of significant value to agriculture [58]. The completion of endosperm development in the absence of fertilization or any paternal genetic contribution represents a rare and pivotal trait for elucidating and engineering true apomixis in crop species [59]. Autonomous apomixis is further relevant for evolutionary and ecological questions because it enables reproduction without any pollen transfer and thus enhances colonization abilities. Plants with autonomous apomixis are overrepresented in cases of geographical parthenogenesis, where apomicts have much larger distribution areas than sexuals [60].

In this study on reproductive phenology, flower and seed analysis using staged seeds and single-seed flow cytometry in *L. algarvense* and *L. daveaui* triploid, self-sterile plants, we have determined phenological developmental stages and morphological markers for the seed coat. Moreover, we found autonomous apomixis with pollen-independent endosperm formation, the transient presence of stage-dependent endosperm nuclei, and earlier initiation of endosperm formation prior to embryo development in immature seeds.

### 4.1. Ploidy Level, Flower and Seed Production in Triploid Limonium Species

Genome size measurements by flow cytometry confirmed a diploid genome for *L. ovalifolium* and a triploid genome for both *L. daveaui* and *L. algarvense*. Previous data have shown that *L. ovalifolium* has 2*n* = 2*x* = 16 chromosomes [16,21] and *L. algarvense* 2*n* = 3*x* = 24, 25, 26 chromosomes [19,21,22,26]. In this study, we found that the studied *L. daveaui* plants had 2*n* = 3*x* = 25 chromosomes.

Several studies have focused on the reproductive development of *Limonium* species. Results on pollen fertility revealed a moderate to high frequency (60.5–93%) in the diploid species [16,19], while triploid *L. algarvense* had very low pollen germination frequencies, varying from 0.8% to 8.2% [19]. In diploid *L. ovalifolium*, estimations of seed set per inflorescence have described more than a hundred seeds in natural populations, with a mean germination rate of 63% [16]. Similarly, despite the low pollen germination, *L. algarvense* plants produce a high percentage of approximately 150 seeds per inflorescence, with a moderate to high seed germination frequency of 65% [19].

In this work, we found differences in flower and seed production among the studied triploid species. Seed production per flower was significantly higher in *L. algarvense* than in *L. daveaui* (Figure 3), meaning a higher percentage of empty flowers in which no seed was formed in the latter species. Therefore, a higher rate of seed abortion occurs in *L. daveaui* than in *L. algarvense*. Differences in seed formation could be related to differences inherent to each species and its origin. Nevertheless, non-extant, or population entities that could be considered as parents of these extant triploid species are unknown. Assumptions on the polyploid apomicts’ parentage are attributed to different combinations of basic chromosome numbers, in which polyploid taxa originated from different combinations of basic numbers *x* = 8 and *x* = 9 are apomicts [21]. However, this hypothesis has not yet been tested.

In *Taraxacum*, a high percentage of undeveloped seeds was observed in a cross-experiment between related diploid sexuals and triploid apomicts, with apomictic hybrids showing partial seed set caused by semi-sterility [61]. A high percentage of sterile florets with aborted seeds was also found in barley mutants with near-normal endosperm development to almost absent [62]. In *Paspalum*, hybrids obtained from intraspecific crosses exhibited a very low seed set due to deviations in the 2:1 maternal to paternal genome contribution required for normal endosperm development, embryo growth and viable seed production [63]. Alternatively, differences in terms of flowering opening in relation to the start of seed formation could be present among the *Limonium* triploid species studied, despite the same seed stage. Further studies on triploid *Limonium* species should be conducted to understand the factors influencing flower fertility and seed production.

### 4.2. Floral Heteromorphisms and Autonomous Apomixis

*Limonium* plants are characterized by a polymorphic sexual system associated with striking flower polymorphisms linked to a sporophytic self-incompatibility system [15,20,36,37]. Our results on floral heteromorphisms revealed that all plants had the pollen–stigma combination B (papillate type stigma and microreticulate pollen) and were self-sterile [20,38]. *Limonium ovalifolium* natural populations have dimorphic pollen-stigma plants which reproduce through outcrossing [15,21]. In the triploid plants studied, immature inflorescences were covered with fine mesh bags prior to anthesis, and because plants were self-sterile (no emasculation required), seeds were formed through apomixis and with pollen-independent endosperm formation. Thus, for both *L. daveaui* and *L. algarvense*, autonomous apomixis was confirmed. As found in other species, apomicts with autonomous endosperm formation produce pollen with low viability [64,65] and may even exhibit male sterility, like in *L. multiflorum* [16]. This is the case of the studied plants for which there is no selective pressure for self-sterility, as pollen functionality is not needed at all, unlike in pseudogamous apomicts [66,67]. Autonomous apomixis has been described in several species and experimental hybrids among families, particularly in Asteraceae, Brassicaceae and Plumbaginaceae [6]. Frequently, autonomous endosperm formation is associated with diplospory [68] as in triploid apomicts (e.g., in Asteraceae like *Erigeron* [69], *Hieracium* [70], *Taraxacum* [61]), and in the studied *Limonium* species. In *Taraxacum* and *Chondrilla* autonomous apomicts, new populations can start from a single colonist and enhance their establishment in remote areas [71].

Flow cytometry seed screening has been demonstrated to allow the determination of reproductive pathways in seed formation [51]. However, ssFCSS patterns are difficult to interpret in autonomous apomicts because the 1:2 embryo:endosperm ratio can mimic G2 peaks of mitotically dividing embryo cells in histograms. *Taraxacum* and *Hieracium* autonomous apomicts are extreme examples of this condition, having lost the normal requirement for paternal contribution to the endosperm [72]. The same is observed in some *Limonium* diploid and tetraploid species, where the endosperm is rapidly consumed [16], as also found in Asteraceae [10]. This is rare in most other families due to deviations from the optimal 2:1 maternal to paternal genome contributions in endosperm [6].

In this study, the diploid sexual *L. ovalifolium* seeds presented histograms with a ratio of embryo to endosperm DNA content of 2C:4C and histograms with a single 2C embryo peak. Reproductive studies revealed that diploid *L. ovalifolium* forms meiotically reduced tetrasporic embryo sacs of *Gagea ova* (most common), *Adoxa* and *Drusa* types [15]. Meiotically unreduced, diplosporic (apomictic) embryo sacs of *Rudbeckia* type were occasionally observed in diploid species (*L. ovalifolium*), but formed exclusively in male sterile tetraploid *L. multiflorum* plants in which autonomous development occurs [15]. The peak index (PI) of 2 produced by the 2C:4C ratio in *L. ovalifolium* developing seeds (stage II) indicates a double fertilization between a reduced egg cell and one sperm cell [1(m)+1(p)], and three polar nuclei with the other sperm cell [1(m)+1(m)+1(m)+1(p)] (Table 3). This is associated with a *Gagea ova* embryo sac [6]. The observation of a single 2C embryo peak with no detectable endosperm peak could result from a meiotically unreduced diplosporic (apomictic) embryo sac of *Rudbeckia* type, with no fertilization [15]. However, since this pattern was only seen in mature seeds (stage III), the absence of endosperm is stage-dependent, because at this stage, the endosperm was already consumed.

In triploid species, ssFCSS allowed the detection of three embryo and endosperm patterns: (1) a single triploid embryo peak, (2) two peaks corresponding to an embryo and an endosperm with a doubled ploidy level, and (3) a single hexaploid endosperm peak. Earlier investigations in triploid facultative apomict *Limonium* species have described diplosporous *Ixeris*-type embryo sacs with non-reduced gametes [14]. The studied plants showed autonomous apomixis with a ratio of embryo to endosperm DNA content of 3C:6C and a PI of 2 in stage II seeds. The pattern observed indicates an unreduced embryo [3(m)], and unfertilized and unreduced three polar nuclei (3Cm+3Cm; Table 3). This is caused by the absence of the paternal genome in seed development as observed in Asteraceae [72]. A cross-experiment between diploid sexual and triploid apomictic *T. officinale* lines revealed autonomous endosperm in triploid hybrids, with a 3C embryo peak and a lower 6C endosperm peak detected by FCSS analysis [11,49]. In experimental hybrids within and between diploid and triploid species from the genus *Crataegus* (Rosaceae), FCSS histogram patterns enabled the detection of different reproductive modes. Namely, seeds with a 3C embryo and a 6C endosperm, possibly autonomously formed without fertilization by sperm cells in an unreduced megagametophyte [73]. Also, in other predominantly pseudogamous plant genera, rare autonomous endosperm formation has been observed (e.g., in *Ranunculus* [74,75,76]).

Interestingly, in this study, triploid immature seeds (stage I) exhibited only a 6C peak with no detectable 3C embryo peak. Since the triploid plants were self-sterile and leaf flow cytometry confirmed their ploidy level, triploid embryos are expected with no ploidy deviations. However, at stage I, embryos are still too small to be detected; thus, the 6C peak corresponded to the endosperm peak. This observation allowed us to discard the possibility of a G2 embryo peak corresponding to the second lower peak seen in stage II seeds. Also in other apomictic plants, endosperm often grows faster than the embryo, resulting in prominent endosperm peaks without visible or very small embryo peaks (e.g., in *Ranunculus auricomus*; [75,76]). The timing of development is crucial for understanding FCSS patterns.

### 4.3. Stage-Dependent Endosperm Development Patterns in Wrinkled and Shrunken Seeds

Previous flow cytometric seed screening investigations using mature seeds of diploid *L. ovalifolium* and tetraploid *L. multiflorum* revealed a unique DNA peak, corresponding to the embryo peak [16]. These mature seeds were characterized as having one embryo and a residual anucleated endosperm formed exclusively by starch grains [16]. It was hypothesized that at an earlier seed development stage, cells with nuclei might have been present, or that the endosperm could be formed by a low number of nucleated cells [16].

Altered timing of endosperm differentiation can perturb seed development as found in triploid *Oenothera* plants resulting from reciprocal diploid–tetraploid crosses [77]. In a cross-experiment between *Taraxacum* diploid sexual and tetraploid apomicts, FCSS results revealed that some plants formed seeds that failed to undergo parthenogenesis but possessed a hexaploid autonomous endosperm [78].

Our results on *Limonium* mature diploid seeds (stage III) confirmed previous observations [16] and allowed the detection of a single 3C embryo in triploid species. Remarkably, in immature wrinkled seeds at stage I, a DNA peak corresponding to the endosperm with the double ploidy level of the embryo was found, proving that endosperm nucleated cells were present at earlier seed development stages. Shrunken and pinkish stage I seeds, appearing underdeveloped, resembled viable seeds in the phase of seed filling. This phase happens after an initial period of cell division and histodifferentiation, characterized by a constant or almost constant increase in seed dry weight, until the maximum dry weight is reached [79]. Furthermore, the ssFCSS pattern showing a unique 6C endosperm peak was never detected in stage II seeds that were bigger and did not show a wrinkled coat. When comparing the DNA content of 6C peaks among young seed stages, it is possible to detect higher values in stage I seeds than in stage II. Therefore, this result supports the highest number of endosperm nucleated cells at stage I, the loss of endosperm nuclei in stage II seeds that already presented an embryo, and finally, anucleated endosperm stage III seeds. Further, a few shrunken/wrinkled seeds at stage I showed a light brown tegument, typically encountered at later seed stages. It could be hypothesized that these seeds will not undergo further development and abort. Alternatively, at this stage, the number of parthenogenetic embryo cells is still too low to be detected through ssFCSS. Altogether, these differences in seed phenological stages, appearance (rugosity), and coat color can be associated with distinct ssFCSS patterns, and therefore can be viewed as morphological markers of seed development.

## 5. Conclusions

Our study on triploid *L. daveaui* and *L. algarvense* self-sterile plants revealed autonomous endosperm formation and showed significant differences in the number of seeds per plant, when considering the number of flowers produced. Macromorphological characterization of seed appearance, coat color, and reproductive phenology, together with single-seed flow cytometry, revealed three endosperm development stages, according to different embryo and endosperm ploidy patterns. Wrinkled/shrunken seeds at stage I showed nucleated endosperm cells with the corresponding 6C peak; stage II seeds, with a pinkish or light brown coat, presented the 3C embryo: 6C endosperm; and stage III seeds, with a dark brown coat, had a unique 3C embryo peak. Thus, the nucleated endosperm is stage-dependent during seed development, and this tissue initiates prior to embryo formation. Future studies should be conducted to understand the potential of the morphological markers described here, associated with gene expression patterns in *Limonium* apomicts with autonomous endosperm development.

## Figures and Tables

**Table 1:** Pollen-stigma combinations, number of dried flowers per plant, absolute number of seeds per plant, % of seeds and somatic ploidy level of the investigated plants. Mean values for each species are indicated at the bottom of each column. The percentage of seeds was calculated as the number of seeds/number of dried flowers per plant × 100 (%). For the diploid *Limonium ovalifolium* (Lo2009I2CR), the number of seeds per inflorescence is indicated. * Literature [56].

Species	Plant	Flower Morph	Number of Dried Flowers per Plant	% of Seeds (Number of Seeds per Plant)	Chromosome Number and Ploidy Level
*Limonium daveaui*	Ld2023I3	B (self-sterile)	385	27 (104)	2*n* = 25 = 3*x*
	Ld2023I4		190	5.3 (10)	
	Ld2023I2		472	41 (194)	
	Ld2023I4s		552	27.5 (152)	
	Ld2023I5		1103	10.3 (114)	
	Ld2023I5cs		878	13 (114)	
	Mean		596.7	20.7 (114.7)	
*Limonium algarvense*	La2023I3	B (self-sterile)	361	54.3 (196)	2*n* = 25 = 3*x*
	La2023I4		253	53.8 (136)	
	La2023I5		437	32.7 (143)	
	La2023I1		359	62.7 (225)	
	La2023I1s		461	44.5 (205)	
	La2023I1cs		269	44.2 (119)	
	Mean		356.7	48.7 (170.7)	
*Limonium ovalifolium* (Lo2009I2CR *)		B (self-sterile)		~100 seeds	2*n* = 16 = 2*x*

**Table 2:** Results obtained through ssFCSS. Mean values are shown for each of the studied *Limonium* species.

	Sample Fluorescence	CV	Ratio to Standard *	DNA Content (pg)	DNA Ploidy Level	Peak Index
*Limonium ovalifolium*	8562.92	3.80	0.39	3.53	2*n* = 2*x*	1.97
	16,785.84	3.72	0.76	6.94	2*n* = 4*x*	
*Limonium daveaui*	12,649.28	3.35	1.48	5.23	2*n* = 3*x*	1.96
	24,756.19	2.98	2.90	10.23	2*n* = 6*x*	
*Limonium algarvense*	13,907.45	3.63	1.63	5.75	2*n* = 3*x*	1.95
	27,085.62	3.15	3.17	11.20	2*n* = 6*x*	

The following data are given for each species: sample fluorescence (median), coefficient of variation (CV, %), fluorescence ratio relative to standard, inferred DNA content and ploidy level, peak index (endosperm tissue ploidy/embryonic tissue ploidy). * As standard, *Pisum sativum* was used to confirm ploidy calculations of the diploid *Limonium ovalifolium*, while single seeds of this species were used to infer the DNA content of the triploid species.

**Table 3:** Reproductive pathways of diploid *Limonium ovalifolium* and triploids *Limonium algarvense* and *Limonium daveaui*.

Ploidy	Reproduction Mode	Embryo sac Type	Genome Contribution to Embryo/Endosperm			PI	Sperm Nuclei Contribution to Endosperm
			Embryo (Cx)	Endosperm (Cx)	Em:End		
Diploid *	Sexual	*Gagea ova*	1(♀) + 1(♂)	3(♀) + 1(♂)	2C:4C	2.0	1
	Sexual	*Adoxa*, *Drusa*	1(♀) + 1(♂)	2(♀) + 1(♂)	2C:3C	1.5	1
	Apomictic	*Rudbeckia*	2(♀)	2(♀)	2C:2C	1.0	0
Triploid	Autonomous apomictic	*Rudbeckia*	3(♀)	3(♀) + 3(♀)	3C:6C	2.0	0

Cx reflects ploidy based on DNA content; ♀, maternal genome contribution (m); ♂, paternal genome contribution (p); PI, peak index. * Literature [15].

## Data Availability

The original contributions presented in this study are included in the article. Further inquiries can be directed to the corresponding author.
